# Health-Related Quality of Life of Individuals with Physical Disabilities in Childhood

**DOI:** 10.3390/children12030365

**Published:** 2025-03-15

**Authors:** Chris Church, Sana Patil, Stephanie Butler, Freeman Miller, Jose J. Salazar-Torres, Nancy Lennon, M. Wade Shrader, Maureen Donohoe, Faithe Kalisperis, W. G. Stuart Mackenzie, Louise Reid Nichols

**Affiliations:** Department of Orthopaedics, Nemours Children’s Health, Wilmington, DE 19803, USA; chris.church@nemours.org (C.C.); sana.patil@nemours.org (S.P.); freeman.miller@gmail.com (F.M.); jose.salazar@nemours.org (J.J.S.-T.); nancy.lennon@nemours.org (N.L.); wade.shrader@nemours.org (M.W.S.); reenee.donohoe@nemours.org (M.D.); faithe.kalisperis@nemours.org (F.K.); stuart.mackenzie@nemours.org (W.G.S.M.); reid.nichols@nemours.org (L.R.N.)

**Keywords:** health-related quality of life, arthrogryposis, achondroplasia, Morquio syndrome, cerebral palsy

## Abstract

**Background**: The use of patient-reported outcomes is essential to understand and manage health-related quality of life (HRQOL) in youth with lifelong disabilities. This study evaluated HRQOL in youth with physical disorders and examined its relationship with mobility. **Methods:** We conducted an IRB-approved retrospective study in which we administered the parent-reported Pediatric Outcomes Data Collection Instrument (PODCI) and Gross Motor Function Measure section D (GMFM-D) to ambulatory youth aged 2–18 years with cerebral palsy (CP; Gross Motor Function Classification System II; *n* = 258), arthrogryposis (*n* = 138), achondroplasia (*n* = 102), and Morquio syndrome (*n* = 52) during clinical visits to a gait lab. The PODCI has two validated versions, child and adolescent, that assess perceptions about mobility, happiness, and pain. Differences in HRQOL between diagnostic groups, between age groups, and compared with non-disabled youth were examined using non-parametric tests. The relationship between GMFM-D and PODCI scores was analyzed with Pearson’s correlations. **Results:** Both age cohorts within all diagnosis groups demonstrated higher pain and lower mobility compared with non-disabled youth (*p* < 0.015). Happiness was lower for both age groups with CP and arthrogryposis, and for the child group with Morquio syndrome compared with non-disabled youth (*p* < 0.002). In diagnostic groups in both age spans, Global Function was higher (*p* < 0.0001) for those with achondroplasia compared with other groups. Despite functional differences, there were no significant differences between diagnostic groups in pain scores (*p* > 0.10). Happiness was lower in the group with CP compared with that with achondroplasia (*p* = 0.01). GMFM-D was related to PODCI mobility scores for all diagnoses *(r* = 0.31 to 0.79, *p* < 0.03) but was not correlated with happiness (*r* = −0.16 to 0.092; *p* > 0.14); GMFM-D and PODCI pain scores were associated only for the child group with achondroplasia (*r* = 0.355; *p* < 0.001). **Conclusions:** Significant limitations in HRQOL are present in youth with physical disabilities. Pain levels were higher than those of non-disabled youth, but pain was not related to lower motor function. Happiness was not related to gross motor function, suggesting the need to examine other factors when mental health concerns are present in youth with disabilities.

## 1. Introduction

Youth with physical disabilities commonly undergo a variety of evidence-based medical treatments, surgeries, and therapies aimed to improve motor function and comfort in their daily lives. However, personal experience and satisfaction with life, or health-related quality of life (HRQOL), is not well documented for those with physical disabilities in childhood [1]. HRQOL is defined as “how well a person functions in their life and his or her perceived well-being in physical, mental, and social domains of health” [2]. The importance of assessing HRQOL in childhood conditions is widely supported in previous research [3,4]. Performing this provides the context for creating a holistic approach to managing the childhood disorders described in the World Health Organization’s International Classification of Function, Disability, and Health: Children and Youth [5]. The Pediatric Outcomes Data Collection Instrument (PODCI) is a well-validated and reliable tool to evaluate HRQOL among youth with physical disabilities, including cerebral palsy, arthrogryposis, congenital upper limb anomalies, hypermobility, and skeletal dysplasia [6,7,8,9,10,11].

While it is important to measure HRQOL, it is also important to consider that it is only part of the concept of quality of life. Quality of life is a complex topic referring to the conceptions of goodness of life and subjective well-being that can be impacted by a variety of factors including health [12]. Narrowing the consideration of quality of life to health-related issues has been criticized due to lack of attention to other factors including socioeconomic, social, and cultural factors [12].

There are many musculoskeletal and neuromotor disorders that affect physical function and likely HRQOL in youth [13]. Cerebral palsy (CP) is one of the most common and is caused by damage to a developing brain resulting in impaired movement and posture [14]. Studies assessing HRQOL have found that youth with CP have the lowest reported physical functioning and psychosocial health compared with nearly 30 other chronic conditions [15,16]. Achondroplasia is the most common type of skeletal dysplasia and involves slow bone growth, short stature, hypermobile joints, spinal stenosis, and bony malalignment [17]. Morquio syndrome, a less common type of skeletal dysplasia, also presents with short stature, spine deformity, and bony malalignment as well as serious systemic issues associated with insufficient breakdown of glycosaminoglycans [18]. Separate studies have found that children with achondroplasia and Morquio syndrome experience significant negative impacts on social and emotional well-being [18,19]. Arthrogryposis is a condition characterized by contractures in two or more joints of the body and commonly presents with muscle weakness and limitations in functional mobility [20,21]. Studies of HRQOL in youth with arthrogryposis find that they have more limited functional mobility compared with non-disabled peers but no differences in happiness [11,22].

Despite a recent increase in studies examining HRQOL among youth with physical disabilities, the data remain limited. There is currently little information on the relationship between motor function and quality of life. A study of children with CP found no association between Gross Motor Function Classification System (GMFCS) levels and quality of life as measured by several outcome tools [23]. A variety of factors have been proposed as being related to HRQOL in children with physical disabilities, including child (behavior, severity of disability, and co-morbidities), caregiver (stress, marital status, socioeconomic), and environmental factors (school, care support) [24].

Understanding HRQOL and the factors that impact youth with disabilities is essential to inform clinical practice, guide treatment plans, and set expectations. The purpose of this study was threefold: first, to compare HRQOL in youth with four common physical disabilities to non-disabled youth using the PODCI; second, to compare PODCI scores between diagnoses and between younger (children aged 2–10 years) and older (adolescents aged 11–18 years) age groups; and third, to evaluate the association between functional mobility using Gross Motor Function Measure section D (GMFM-D) and PODCI scores. We hypothesize that HRQOL will be lower in children with physical disabilities than non-disabled youth, that HRQOL will be different in younger and older children, and that there will be a significant association between functional mobility and HRQOL.

## 2. Materials and Methods

This institutional review board-approved retrospective study utilized information from clinical visits to our institution’s gait laboratory from January 2004 until May 2023. Parents of the participants completed the PODCI questionnaires [6], and physical therapists administered the GMFM-D [25] during these visits. Participants were ambulatory youth aged 2–18 years with a diagnosis of either CP, achondroplasia, arthrogryposis, or Morquio syndrome. These groups were selected based on their limitations in HRQOL based on clinical experience and the frequency in which they were seen in our clinics. Only youth with CP functioning at GMFCS level II [26] were included. All other GMFCS levels were excluded to decrease variability within the diagnosis group and to maintain a cohort that could be similarly compared with the other diagnoses. Participants were also excluded if they were missing PODCI scores from two or more domains, if they had undergone orthopedic surgery within the year prior to completion of the PODCI, or if only self-reported PODCI scores were available. If a participant had multiple gait lab visits with complete PODCI data, the visit when the participant was closest to age 5 and/or 15 years was selected for analysis.

The PODCI was developed by the Pediatric Orthopaedic Society of North America, the American Academy of Orthopaedic Surgeons, the American Academy of Pediatrics, and Shriner’s Hospitals to assess HRQOL, including comfort, happiness, and everyday performance in youth with orthopedic issues aged 2–18 years. The PODCI evaluates HRQOL using sub-scores from five domains [6]. Three domains assess physical function including upper extremity and physical function, Transfer and Basic Mobility, and Sports and Physical Functioning, and two assess happiness and pain/comfort. A Global Function score averages the upper extremity and physical function, Transfer and Basic Mobility, Sports and Physical Functioning, and pain/comfort domains [27]. There are two versions of the instrument based on age, namely child (age 2–10 years) and adolescent (age 11–18 years) versions that investigate parents’ perceptions of the child or adolescent during the last week [28].

The Gross Motor Function Measure-88 is a standardized, objective measure of physical function [25]. Section D focuses on standing and examines 13 activities including independent standing, single-leg balance, transitioning from sitting to standing, and transitioning from the floor to standing. The test is administered by a physical therapist who rates the ability to complete the activities on a scale from 0 to 3 [29,30].

A *t*-test was used to compare mean PODCI scores from each age band within each diagnosis to normative parent-reported scores for non-disabled youth [31]. As the collected data were not normally distributed, non-parametric tests were used for the remaining analyses. To determine if there were differences in GMFM-D and PODCI scores between diagnoses, medians for each diagnosis were compared using the Kruskal–Wallis test. To analyze pairwise comparisons for PODCI and GMFM-D scores between each of the diagnoses, the Wilcoxon rank-sum test was conducted with Bonferroni corrections for multiple comparisons to minimize the rate of type II error. Median PODCI and GMFM-D scores were compared between child and adolescent age groups in each diagnosis using the Wilcoxon rank-sum test. Lastly, Pearson’s correlations were conducted between PODCI subdomain scores and GMFM-D scores to examine the relationship between HRQOL and physical function.

## 3. Results

Data from 550 patients, ages 2–18, were analyzed (Table 1). CP was the largest group (*n* = 258) and had a total mean age that was statistically higher (*p* < 0.007) than all other diagnosis groups. The next largest group was arthrogryposis (*n* = 138), followed by achondroplasia (*n* = 102) and Morquio syndrome (*n* = 52). Published PODCI norms from non-disabled youth were sampled from 1762 children (ages 2–10 years) and 3538 adolescents (ages 11–18 years) [31].

Youth within the diagnosis groups were found to have lower PODCI scores compared with non-disabled youth. Significant differences were found between both the child and adolescent age groups in each diagnosis and non-disabled youth in all PODCI domains (*p* < 0.02) except happiness (Table 2 and Table 3). The child and adolescent groups with achondroplasia and the adolescent group with Morquio syndrome had no significant differences in the happiness domain compared with non-disabled youth. The child groups with CP, arthrogryposis, and Morquio syndrome exhibited larger ranges of scores in the functional domains compared with the child group with achondroplasia. Across all diagnoses and age groups, larger ranges were observed in the pain/comfort and happiness domain scores compared with other domains.

Comparisons between diagnostic groups in PODCI and GMFM-D scores are detailed in Table 4. The only significant differences found between diagnosis groups for pain/comfort and happiness scores were between the child groups with CP and achondroplasia (*p* = 0.01). The child group with achondroplasia also had significantly higher scores on PODCI domains related to functional mobility (upper extremity and physical function, Sports and Physical Functioning, Transfer and Basic Mobility) and higher GMFM-D scores (*p* < 0.0001) than the child groups in all other diagnoses. The child groups with CP and arthrogryposis scored similarly on all PODCI domains and the GMFM-D, with no significant differences (*p* > 0.05).

Comparisons between the adolescent and child groups within each diagnosis in PODCI and GMFM-D scores are detailed in Table 5. The achondroplasia diagnosis had the most significant differences between the child and adolescent age groups, with adolescents having significantly higher upper extremity and physical function, Transfer and Basic Mobility, and Global Functioning scores but lower happiness scores (*p* < 0.01) compared with the child group. The child and adolescent groups with CP had significantly different scores (*p* < 0.05) in two of the five PODCI domains (Transfer and Basic Mobility, happiness). No significant differences were seen between the child and adolescent groups with Morquio syndrome in any PODCI domain, but GMFM-D scores were higher in the child group compared with the adolescent group (*p* = 0.023). Across all diagnoses, there were no significant differences in pain/comfort scores between the child and adolescent groups. The child groups with CP, arthrogryposis, and achondroplasia had higher happiness scores than their adolescent counterparts (*p* < 0.05). In these same three diagnostic groups, Transfer and Basic Mobility scores were higher in adolescent groups compared with child groups (*p* < 0.01). Like the trend in youth with physical disabilities, the non-disabled adolescent reference group had lower happiness scores compared with the non-disabled child reference group (*p* < 0.0001).

Finally, in each diagnosis, GMFM-D scores were weakly to moderately correlated (*p* < 0.05) with the functional PODCI domain scores (upper extremity and physical function, Transfer and Basic Mobility, and Sports and Physical Functioning) and Global Function scores (Table 6). There was no correlation between PODCI happiness scores and GMFM-D scores in any diagnoses. Pain/comfort scores were weakly correlated with GMFM-D scores in the achondroplasia group (*p* < 0.001) but not for any of the other diagnosis groups.

## 4. Discussion

This study found significantly lower HRQOL among youth with common physical disabilities in childhood compared with non-disabled youth. Our work is consistent with previous research showing limited functional mobility and higher pain among children with these same diagnoses [19,32,33,34,35,36]. GMFM-D correlated with parent-reported assessments of functional mobility but not with Pain and Comfort. Although our study showed that youth with physical disabilities had more pain than non-disabled youth, youth with more gross motor limitations do not have more pain.

Our study also found that happiness was lower compared with non-disabled youth, but level of happiness was not related to gross motor function. This suggests a need to evaluate other factors that might contribute to the psychological well-being of youth with physical disabilities when concerns arise. Additionally, when children and adolescents with physical disabilities report low happiness scores on quality-of-life tools, clinicians should not assume that this score is related to their disability. These results are supported by work published by Lennon et al. [1], who found no significant differences between youth with CP functioning at different GMFCS levels in the happiness domain of the PODCI. Our study differs in that we found significantly lower happiness scores in youth with CP compared with non-disabled youth, while Lennon et al. [1] found no differences in PODCI happiness scores between youth with CP and non-disabled youth. The difference might be explained by self [1] vs. proxy reports (our study). Studies have found differences between parent-reported and adolescent self-reported scores, showing that parent-reported scores tend to be lower than self-reported scores [1,37]. Other studies suggest that youth with arthrogryposis have similar levels of psychosocial well-being or happiness compared with non-disabled youth [22,38,39], but the current study demonstrates lower levels of happiness in both children and adolescents with arthrogryposis compared with non-disabled youth peers. Like our findings of lower levels of happiness in the Morquio syndrome group compared with non-disabled youth, Hendriksz et al. [18] report findings of youth with Morquio syndrome expressing difficulty establishing social relationships, with more than half of those surveyed expressing psychological symptoms (depression or other).

Our results show that parental reports of happiness differ between the child and adolescent groups. In non-disabled youth, adolescence is recognized as a transitional time during which reorganization of the circuity in the brain takes place, leaving adolescents more vulnerable to poorer mental health [40]. Additionally, adolescence is a critical and challenging period of development in which young people undergo physical and emotional growth and the development of personal and sexual identity [41]. Our results of lower reported happiness during adolescence compared with childhood in those with physical disabilities follows this same pattern. While it is understood that motor skills generally improve from childhood to adolescence [42], less is known about how the physiological maturation process that occurs during adolescence affects those with physical disabilities. Previous work by Canha et al. [43] found that among children with physical disabilities aged 10–20 years, older individuals were more likely to rate their health status as poor, while younger children were likely to rate their health status as excellent. This transformation in self-awareness of health with age might provide an explanation for changing happiness levels throughout the life of a child with a disability. Psychosocial care for youth with physical disabilities needs further study to ensure the needs of children are identified and addressed. Our findings support the need for child- and family-centered, holistic care focused on maximizing HRQOL. Utilization of parent (or caregiver)-reported health measures for youth with physical disabilities is vital for clinicians to develop treatment plans and set goals that have physical, emotional, and psychosocial needs at the center of plans of care.

Some limitations of this work include the differences in sample sizes and ages of the diagnostic groups. The number of participants with Morquio syndrome was about five times smaller than the number in the largest group (CP) due to the rarity of the syndrome. This likely attenuated the power of statistical findings for the Morquio syndrome group. The cross-sectional nature of this study prevents true analysis of changes experienced with age. Additionally, the CP group was statistically older than all other diagnosis groups, which may have affected PODCI or GMFM scores. To compare child and adolescent PODCI scores, our study used only parent reports for analysis. These can differ from self-assessment but are still valid measures of HRQOL, particularly in cases where intellectual disability or younger age might preclude the child’s ability to answer questionnaires. The retrospective review of data collected as part of clinical evaluation may also impact results, and prospective study would be beneficial. The GMFM-D is well validated for use in children with CP; however, its validity and reliability for all childhood conditions is unknown. It has been used in children with Down syndrome, arthrogryposis, and traumatic brain injuries [22,29,30]. *T*-tests had to be used in statistical comparisons involving PODCI scores for non-disabled individuals, as only means and standard deviations were publicly available. Finally, we acknowledge that newer, diagnosis-specific instruments may be better suited to measure HRQOL in today’s clinical environment rather than the PODCI.

Further study is needed to assess the factors that influence the psychosocial well-being of youth with physical disabilities. The literature has focused on the treatment of functional mobility in children with disabilities but given the lack of relationship between mobility and happiness observed in this study, additional factors should be considered to positively impact mental health. To further investigate how age might affect perspectives over the course of childhood, a prospective, longitudinal cohort study is recommended.

## 5. Conclusions

Youth with these four physical disabilities present with lower HRQOL compared with non-disabled youth and report higher pain. Despite wide disparity in motor function, differences in happiness were minimal and did not correlate with functional mobility. The findings from our study emphasize the importance of individualized care when evaluating and treating both the physical and psychosocial needs of youth with physical disabilities. The use of patient- and family-reported HRQOL measures is a first step to better understand the factors that contribute to physical and psychosocial function and are an important tool needed to create clinical care plans that effectively address all of the child’s and family’s needs.

## Figures and Tables

**Table 1:** Demographic information for the four orthopedic disorders and non-disabled children.

	Cerebral Palsy	Arthrogryposis	Achondroplasia	Morquio Syndrome	Non-Disabled Youth
Total, *n*	258	138	102	52	5300
Total mean age, y	12.1 ± 3.7	9.9 ± 4.5	10.5 ± 3.8	9.4 ± 3.5	
Children, *n*	102	76	54	37	1762
Child mean age, y	8.2 ± 2.0	6.2 ± 2.1	7.5 ± 2.1	7.5 ± 1.6	
Adolescents, *n*	156	62	48	15	3538
Adolescent mean age, y	14.6 ± 2.0	14.3 ± 1.9	14.0 ± 2.1	14.1 ± 2.2	

**Table 2:** Means and standard deviations (SDs) for the Pediatric Outcomes Data Collection Instrument (PODCI) domains and global scores for children and adolescents with cerebral palsy, arthrogryposis, achondroplasia, and Morquio syndrome.

	Non-Disabled Youth		Cerebral Palsy				Arthrogryposis				Achondroplasia				Morquio Syndrome			
			Child		Adolescent		Child		Adolescent		Child		Adolescent		Child		Adolescent	
	Child	Adolescent	Mean	*p* Value	Mean	*p* Value	Mean	*p* Value	Mean	*p* Value	Mean	*p* Value	Mean	*p* Value	Mean	*p* Value	Mean	*p* Value
UEPF	92 ± 11	99 ± 5	72 ± 19	*	75 ± 21	*	65 ± 24	*	84 ± 16	*	81 ± 15	*	92 ± 9	*	62 ± 19	*	70 ± 23	*
SPF	90 ± 12	94 ± 11	52 ± 16.	*	49 ± 18	*	54 ± 22	*	50 ± 21	*	68 ± 16	*	73 ± 17	*	47 ± 18	*	40 ± 20	*
TBM	98 ± 6	99 ± 5	81 ± 14	*	85 ± 13	*	77 ± 20	*	87 ± 14	*	88 ± 12	*	95 ± 9	*	77 ± 16	*	76 ± 19	*
Happiness	90 ± 14	81 ± 18	80 ± 20	*	73 ± 23	*	82 ± 22	**	67 ± 26	*	88 ± 14	0.378	78 ± 18	0.211	75 ± 23	*	75 ± 22	0.249
PC	92 ± 14	89 ± 17	79 ± 23	*	77 ± 23	*	82 ± 20	*	73 ± 26	*	73 ± 22	*	79 ± 22	**	68 ± 23	*	71 ± 28	***
GF	93 ± 8	95 ± 7	71 ± 13	*	71 ± 14	*	69 ± 17	*	73 ± 15	*	78 ± 13	*	85 ± 11	*	64 ± 4	*	64± 6	*

Values for each of the orthopedic conditions were compared with non-disabled children using *t*-tests. Shaded cells indicate nonsignificant differences in means between those with orthopedic conditions and non-disabled youth (mean ± SD). * = <0.001, ** = <0.01, *** = <0.05 indicate statistically significant differences. GF, Global Functioning; PC, Pain and Comfort; SPF, Sports and Physical Functioning; TBM, Transfer and Basic Mobility; UEPF, Upper Extremity Physical Function.

**Table 3:** Means, standard deviations, and minimum and maximum scores for the Pediatric Outcomes Data Collection Instrument (PODCI) domains and global score for children and adolescents with cerebral palsy, arthrogryposis, achondroplasia, and Morquio syndrome.

	Cerebral Palsy						Arthrogryposis						Achondroplasia						Morquio Syndrome					
	Child			Adolescent			Child			Adolescent			Child			Adolescent			Child			Adolescent		
	Mean	Min	Max	Mean	Min	Max	Mean	Min	Max	Mean	Min	Max	Mean	Min	Max	Mean	Min	Max	Mean	Min	Max	Mean	Min	Max
UEPF	72 ± 19	21	100	75 ± 21	8	100	65 ± 24	8	100	84 ± 16	25	100	81 ± 15	25	100	92 ± 9	67	100	62 ± 19	25	96	70 ± 23	25	100
SPF	52 ± 16	18	94	49 ± 18	13	91	54 ± 22	0	95	50 ± 21	5	94	68 ± 16	29	98	73 ± 17	23	100	47 ± 18	9	85	40 ± 20	5	74
TBM	81 ± 14	35	100	85 ± 13	35	100	77 ± 20	12	100	87 ± 14	41	100	88 ± 12	47	100	95 ± 9	52	100	77 ± 16	38	98	76 ± 19	41	97
Happiness	80 ± 20	0	100	73 ± 23	0	100	82 ± 22	0	100	67 ± 26	5	100	88 ± 14	45	100	78 ± 18	35	100	75 ± 23	11	100	75 ± 22	40	100
PC	79 ± 23	0	100	77 ± 23	7	100	82 ± 20	24	100	73 ± 26	7	100	73 ± 22	30	100	79 ± 22	18	100	68 ± 23	15	100	71 ± 28	22	100
GF	71 ± 13	25	99	71 ± 14	35	98	69 ± 17	16	97	73 ± 15	33	96	78 ± 13	51	99	85 ± 11	44	99	64 ± 14	33	91	64 ± 16	33	91

GF, Global Functioning; PC, Pain and Comfort; SPF, Sports and Physical Functioning; TBM, Transfer and Basic Mobility; UEPF, Upper Extremity Physical Function.

**Table 4:** Medians, confidence intervals, and *p* values for pairwise comparisons between diagnoses for the Pediatric Outcomes Data Collection Instrument (PODCI) and scores for section D of the Gross Motor Function Measure-88 (GMFM-D).

	Comparison	Cerebral Palsy		Arthrogryposis		Achondroplasia		Morquio Syndrome	
		Median	*p* Value	Median	*p* Value	Median	*p* Value	Median	*p* Value
UEPF	vs. CP	79 (3)		79 (4)	1	92 (3)	*	65 (6)	***
	vs. Arthro		1				*		***
	vs. Achond		*		*				*
	vs. MS		***		***		*		
SPF	vs. CP	49 (2)		53 (4)	1	72 (3)	*	46 (5)	0.66
	vs. Arthro		1				*		0.289
	vs. Achond		*		*				*
	vs. MS		0.66		0.289		*		
TBM	vs. CP	86 (2)		87 (3)	1	94 (2)	*	81 (5)	0.089
	vs. Arthro		1				*		0.298
	vs. Achond		*		*				*
	vs. MS		0.089		0.298		*		
Happiness	vs. CP	80 (3)		80 (4)	1	90 (3)	***	80 (6)	1
	vs. Arthro		1				0.087		1
	vs. Achond		***		0.087				0.281
	vs. MS		1		1		0.281		
PC	vs. CP	82 (3)		84 (4)	1	80 (4)	1	72 (7)	0.113
	vs. Arthro		1				1		0.127
	vs. Achond		1		1				0.69
	vs. MS		0.113		0.127		0.69		
GF	vs. CP	72 (2)		73 (3)	1	83 (2)	*	66 (4)	**
	vs. Arthro		1				*		***
	vs. Achond		*		*				*
	vs. MS		**		***		*		
GMFM-D	vs. CP	31 (0.4)		31 (1)	1	39 (0.4)	*	32 (1)	1
	vs. Arthro		1				*		1
	vs. Achond		*		*				*
	vs. MS		1		1		*		

Values were compared using the Wilcoxon rank-sum test with Bonferroni corrections for multiple comparisons. * = <0.001, ** = <0.01, and *** = <0.05 indicate statistically significant differences. Achon, achondroplasia; Arthro, arthrogryposis; CP, cerebral palsy; GF, Global Functioning; MS, Morquio syndrome; PC, Pain and Comfort; SPF, Sports and Physical Functioning; TBM, Transfer and Basic Mobility; UEPF, Upper Extremity Physical Function.

**Table 5:** Means, standard deviations, and *p* values for comparisons between non-disabled youth and medians, confidence intervals, and *p* values for pairwise comparisons between children (2–10 years) and adolescents (11–18 years) for the Pediatric Outcomes Data Collection Instrument (PODCI) and scores for section D of the Gross Motor Function Measure-88 (GMFM-D).

	Non-Disabled Youth			Cerebral Palsy			Arthrogryposis			Achondroplasia			Morquio Syndrome		
	Child	Adolescent	*p* Value	Child	Adolescent	*p* Value	Child	Adolescent	*p* Value	Child	Adolescent	*p* Value	Child	Adolescent	*p* Value
UEPF	92 ± 11	99 ± 5	*	74 (4)	83 (3)	0.117	71 (6)	88 (4)	*	83 (4)	94 (2)	*	61 (6)	71 (12)	0.124
SPF	90 ± 12	94 ± 11	*	50 (3)	49 (3)	0.225	53 (5)	51 (5)	0.405	70 (4)	75 (5)	0.117	46 (6)	43 (11)	0.28
TBM	98 ± 6	99 ± 5	*	83 (3)	88 (2)	**	82 (5)	91 (3)	*	92 (3)	97 (3)	*	81 (5)	83 (10)	0.984
Happiness	90 ± 14	81 ± 18	*	85 (4)	75 (4)	***	90 (5)	70 (7)	*	95 (4)	78 (5)	**	80 (8)	75 (12)	0.815
PC	92 ± 14	89 ± 17	*	87 (5)	81 (4)	0.369	89 (5)	79 (7)	0.0636	71 (6)	87 (6)	0.152	67 (8)	85 (15)	0.549
GF	93 ± 8	95 ± 7	*	71 (3)	73 (2)	0.538	69 (4)	75 (4)	0.113	80 (3)	88 (3)	**	65 (5)	67 (9)	0.96
GMFM-D				31 (1)	31 (0.5)	0.901	29 (2)	33 (2)	**	39 (0.5)	39 (0.6)	**	33 (2)	29 (3)	***

Values were compared using the Wilcoxon rank-sum test. * = <0.001, ** = <0.01, and *** = <0.05 indicate statistically significant differences. GF, Global Functioning; PC, Pain and Comfort; SPF, Sports and Physical Functioning; TBM, Transfer and Basic Mobility; UEPF, Upper Extremity Physical Function.

**Table 6:** Pearson’s correlation coefficients and *p* values for correlational analysis between section D of the Gross Motor Function Measure (GMFM-D) and each of the Pediatric Outcomes Data Collection Instrument (PODCI).

	Cerebral Palsy		Achondroplasia		Arthrogryposis		Morquio	
	*r*	*p* Value	*r*	*p* Value	*r*	*p* Value	*r*	*p* Value
GMFM-D vs. UEPF	0.322	*	0.31	**	0.445	*	0.305	***
GMFM-D vs. TBM	0.466	*	0.598	*	0.793	*	0.54	*
GMFM-D vs. SPF	0.389	*	0.512	*	0.572	*	0.568	*
GMFM-D vs. PC	0.016	0.80039	0.355	*	0.164	0.054337	−0.224	0.110854
GMFM-D vs. Happiness	0.092	0.14288	−0.005	0.961831	0.029	0.74129	−0.162	0.249947
GMFM-D vs. GF	0.373	*	0.526	*	0.648	*	0.361	**

* = <0.001, ** = <0.01, and *** = <0.05 indicate statistically significant differences. GF, Global Functioning; PC, Pain and Comfort; SPF, Sports and Physical Functioning; TBM, Transfer and Basic Mobility; UEPF, Upper Extremity Physical Function.

## Data Availability

Data are available upon request from the corresponding author. The data are not publicly available due to institution’s privacy restrictions.

## Abbreviations

The following abbreviations are used in this manuscript:

Achond	Achondroplasia
Arthro	Arthrogryposis
CP	Cerebral Palsy
GMFCS	Gross Motor Function Classification System
GMFM-D	Gross Motor Function Measure Dimension D
HRQOL	Health-Related Quality Of Life
PODCI	Pediatric Outcomes Data Collection Instrument
SPF	Sports And Physical Functioning
TBM	Transfer And Basic Mobility
UEPF	Upper Extremity And Physical Function

## References

[1] Lennon N., Kalisperis F., Church C., Niiler T., Miller F., Biermann I., Davey J., Sees J.P., Shrader M.W. (2024). Self-Reported Health-Related Quality of Life in Adolescents and Young Adults with Cerebral Palsy. J. Pediatr. Orthop..

[2] Hays R.D., Reeve B.B., Killewo J., Heggenhougen H.K., Quah S.R. (2010). Measurement and Modeling of Health-Related Quality of Life. Epidemiology and Demography in Public Health.

[3] Colver A., Rapp M., Eisemann N., Ehlinger V., Thyen U., Dickinson H.O., Parkes J., Parkinson K., Nystrand M., Fauconnier J. (2015). Self-Reported Quality of Life of Adolescents with Cerebral Palsy: A Cross-Sectional and Longitudinal Analysis. Lancet.

[4] Livingston M.H., Rosenbaum P.L., Russell D.J., Palisano R.J. (2007). Quality of Life Among Adolescents with Cerebral Palsy: What Does the Literature Tell Us?. Dev. Med. Child Neurol..

[5] World Health Organization (2007). International Classification of Functioning, Disability and Health: Children and Youth Version: ICF-CY. https://iris.who.int/bitstream/handle/10665/43737/9789241547321_eng.pdf;jsessionid=CF5BD782DFB9CBBB613805684EE5464A?sequence=1.

[6] Daltroy L.H., Liang M.H., Fossel A.H., Goldberg M.J. (1998). The POSNA Pediatric Musculoskeletal Functional Health Questionnaire: Report on Reliability, Validity, and Sensitivity to Change. Pediatric Outcomes Instrument Development Group. Pediatric Orthopaedic Society of North America. J. Pediatr. Orthop..

[7] Shotwell C., Moore E.S. (2022). Assessing Reliability and Validity of a Functional Outcome Measure for Adolescents with Hypermobility Spectrum Disorder. Disabil. Rehabil..

[8] McCarthy M.L., Silberstein C.E., Atkins E.A., Harryman S.E., Sponseller P.D., Hadley-Miller N.A. (2002). Comparing Reliability and Validity of Pediatric Instruments for Measuring Health and Well-Being of Children with Spastic Cerebral Palsy. Dev. Med. Child Neurol..

[9] O’Brien A., Bompadre V., Hale S., White K.K. (2014). Musculoskeletal Function in Patients with Mucopolysaccharidosis Using the Pediatric Outcomes Data Collection Instrument. J. Pediatr. Orthop..

[10] Wall L.B., Vuillermin C., Miller P.E., Bae D.S., Goldfarb C.A. (2020). Convergent Validity of PODCI and PROMIS Domains in Congenital Upper Limb Anomalies. J. Hand Surg. Am..

[11] Cachecho S., Boruff J., Wong T., Lacombe F., Dahan-Oliel N. (2021). Psychosocial Wellbeing Among Children and Adults with Arthrogryposis: A Scoping Review. Health Qual. Life Outcomes.

[12] Lin X.J., Lin I.M., Fan S.Y. (2013). Methodological Issues in Measuring Health-Related Quality of Life. Tzu Chi Med. J..

[13] Nievelsteinj R.A.J., Hodler J., Kubik-Huch R.A., von Schulthess G.K. (2021). Non-Traumatic Musculoskeletal Diseases in Children. Musculoskeletal Diseases 2021–2024: Diagnostic Imaging.

[14] Rosenbaum P., Paneth N., Leviton A., Goldstein M., Bax M., Damiano D., Dan B., Jacobsson B. (2007). A Teport: The Definition and Classification of Cerebral Palsy April 2006. Dev. Med. Child Neurol. Suppl..

[15] Pinquart M. (2020). Health-Related Quality of Life of Young People with and Without Chronic Conditions. J. Pediatr. Psychol..

[16] Varni J.W., Limbers C.A., Burwinkle T.M. (2007). Impaired Health-Related Quality of Life in Children and Adolescents with Chronic Conditions: A Comparative Analysis of 10 Disease Clusters and 33 Disease Categories/Severities Utilizing the PedsQL 4.0 Generic Core Scales. Health Qual. Life Outcomes.

[17] Pauli R.M. (2019). Achondroplasia: A Comprehensive Clinical Review. Orphanet J. Rare Dis..

[18] Hendriksz C.J., Lavery C., Coker M., Ucar S.K., Jain M., Bell L., Lampe C. (2014). Burden of Disease in Patients with Morquio A Syndrome: Results from an International Patient-Reported Outcomes Survey. Orphanet J. Rare Dis..

[19] Pfeiffer K.M., Brod M., Smith A., Gianettoni J., Viuff D., Ota S., Charlton R.W. (2021). Assessing Physical Symptoms, Daily Functioning, and Well-Being in Children with Achondroplasia. Am. J. Med. Genet. A.

[20] Bamshad M., Van Heest A.E., Pleasure D. (2009). Arthrogryposis: A Review and Update. J. Bone Joint Surg. Am..

[21] Langston S., Chu A. (2020). Arthrogryposis Multiplex Congenita. Pediatr. Ann..

[22] Church C., McGowan A., Henley J., Donohoe M., Niiler T., Shrader M.W., Nichols L.R. (2020). The 5-Year Outcome of the Ponseti Method in Children with Idiopathic Clubfoot and Arthrogryposis. J. Pediatr. Orthop..

[23] Oeffinger D., Gorton G., Bagley A., Nicholson D., Barnes D., Calmes J., Abel M., Damiano D., Kryscio R., Rogers S. (2007). Outcome Assessments in Children with Cerebral Palsy, Part I: Descriptive Characteristics of GMFCS Levels I to III. Dev. Med. Child Neurol..

[24] Tseng M.H., Chen K.L., Shieh J.Y., Lu L., Huang C.Y., Simeonsson R.J. (2016). Child Characteristics, Caregiver Characteristics, and Environmental Factors Affecting the Quality of Life of Caregivers of Children with Cerebral Palsy. Disabil. Rehabil..

[25] Russell D.J., Rosenbaum P.L., Wright M., Avery L.M. (2002). Gross Motor Function Measure (GMFM-66 & GMFM-88) User’s Manual.

[26] Palisano R., Rosenbaum P., Walter S., Russell D., Wood E., Galuppi B. (1997). Development and Reliability of a System to Classify Gross Motor Function in Children with Cerebral Palsy. Dev. Med. Child Neurol..

[27] Klepper S.E. (2011). Measures of Pediatric Function: Child Health Assessment Questionnaire (C-HAQ), Juvenile Arthritis Functional Assessment Scale (JAFAS), Pediatric Outcomes Data Collection Instrument (PODCI), and Activities Scale for Kids (ASK). Arthritis Care Res..

[28] Greer A.E., Iversen M.D. (2020). Measures of Pediatric Function and Physical Activity in Arthritis. Arthritis Care Res..

[29] Linder-Lucht M., Othmer V., Walther M., Vry J., Michaelis U., Stein S., Weissenmayer H., Korinthenberg R., Mall V., Gross Motor Function Measure-Traumatic Brain Injury Study Group (2007). Validation of the Gross Motor Function Measure for Use in Children and Adolescents with Traumatic Brain Injuries. Pediatrics.

[30] Russell D., Palisano R., Walter S., Rosenbaum P., Gemus M., Gowland C., Galuppi B., Lane M. (1998). Evaluating Motor Function in Children with Down Syndrome: Validity of the GMFM. Dev. Med. Child Neurol..

[31] Hunsaker F.G., Cioffi D.A., Amadio P.C., Wright J.G., Caughlin B. (2002). The American Academy of Orthopaedic Surgeons Outcomes Instruments: Normative Values from the General Population. J. Bone Joint. Surg. Am..

[32] Altiok H., Flanagan A., Krzak J.J., Hassani S. (2019). Quality of Life, Satisfaction with Life, and Functional Mobility of Young Adults with Arthrogryposis After Leaving Pediatric Care. Am. J. Med. Genet. C Semin. Med. Genet..

[33] Cirillo A., Collins J., Sawatzky B., Hamdy R., Dahan-Oliel N. (2019). Pain Among Children and Adults Living with Arthrogryposis Multiplex Congenita: A Scoping Review. Am. J. Med. Genet. C Semin. Med. Genet..

[34] Eriksson E., Hägglund G., Alriksson-Schmidt A.I. (2020). Pain in Children and Adolescents with Cerebral Palsy—A Cross-Sectional Register Study of 3545 Individuals. BMC Neurol..

[35] Hendriksz C.J., Berger K.I., Lampe C., Kircher S.G., Orchard P.J., Southall R., Long S., Sande S., Gold J.I. (2016). Health-Related Quality of Life in Mucopolysaccharidosis: Looking Beyond Biomedical Issues. Orphanet J. Rare Dis..

[36] Makris T., Dorstyn D., Crettenden A. (2021). Quality of Life in Children and Adolescents with Cerebral Palsy: A Systematic Review with Meta-Analysis. Disabil. Rehabil..

[37] Longo E., Badia M., Begoña Orgaz M., Gómez-Vela M. (2017). Comparing Parent and Child Reports of Health-Related Quality of Life and Their Relationship with Leisure Participation in Children and Adolescents with Cerebral Palsy. Res. Dev. Disabil..

[38] Hyer L.C., Carson L.T., Carpenter A.M., Westberry D.E. (2021). Patient-Reported Outcome Measurement Information System (PROMIS) Scores in Pediatric Patients with Arthrogryposis. J. Pediatr. Orthop..

[39] Wall L.B., Vuillerman C., Miller P.E., Bae D.S., Goldfarb C.A., CoULD Study Group (2007). Patient-Reported Outcomes in Arthrogryposis. J. Pediatr. Orthop..

[40] Konrad K., Firk C., Uhlhaas P.J. (2013). Brain Development During Adolescence: Neuroscientific Insights into this Developmental Period. Dtsch. Arztebl. Int..

[41] Christie D., Viner R. (2005). Adolescent Development. BMJ.

[42] Branta C., Haubenstricker J., Seefeldt V. (1984). Age changes in motor skills during childhood and adolescence. Exerc. Sport Sci. Rev..

[43] Canha L., Simões C., Matos M.G., Owens L. (2016). Well-Being and Health in Adolescents with Disabilities. Psicol. Refl. Crít..

